# Comprehensive Analysis of Survival-Related lncRNAs, miRNAs, and mRNAs Forming a Competing Endogenous RNA Network in Gastric Cancer

**DOI:** 10.3389/fgene.2021.610501

**Published:** 2021-03-02

**Authors:** Yanjie Zhao, Heng Zhang, Qiang Ju, Xinmei Li, Yuxin Zheng

**Affiliations:** ^1^School of Public Health, Qingdao University, Qingdao, China; ^2^Department of Blood Transfusion, The Affiliated Hospital of Qingdao University, Qingdao University, Qingdao, China

**Keywords:** gastric cancer, lncRNA, miRNA, mRNA, ceRNA, prognosis

## Abstract

To analyze and construct a survival-related endogenous RNA (ceRNA) network in gastric cancer (GC) with lymph node metastasis, we obtained expression profiles of long non-coding RNAs (lncRNAs), mRNAs, and microRNAs (miRNAs) in GC from The Cancer Genome Atlas database. The edgeR package was used to screen differentially expressed lncRNAs, mRNAs, and miRNAs between GC patients with lymphatic metastasis and those without lymphatic metastasis. Then, we used univariate Cox regression analysis to identify survival-related differentially expressed RNAs. In addition, we used multivariate Cox regression analysis to screen lncRNAs, miRNAs, and mRNAs for use in the prognostic prediction models. The results showed that 2,247 lncRNAs, 155 miRNAs, and 1,253 mRNAs were differentially expressed between the two patient groups. Using univariate Cox regression analysis, we found that 395 lncRNAs, eight miRNAs, and 180 mRNAs were significantly related to the survival time of GC patients. We next created a survival-related network consisting of 59 lncRNAs, seven miRNAs, and 36 mRNAs. In addition, we identified eight RNAs associated with prognosis by multivariate Cox regression analysis, comprising three lncRNAs (AC094104.2, AC010457.1, and AC091832.1), two miRNAs (miR-653-5p and miR-3923), and three mRNAs (C5orf46, EPHA8, and HPR); these were used to construct the prognostic prediction models, and their risk scores could be used to assess GC patients’ prognosis. In conclusion, this study provides new insights into ceRNA networks in GC and the screening of prognostic biomarkers for GC.

## Introduction

Gastric cancer (GC) is the third leading cause of cancer-related mortality worldwide (Venerito et al., 2018). A recent study demonstrated that lymph node metastasis is common among patients with GC (Bravo Neto et al., 2014). Lymphatic metastasis is the leading cause of mortality and a significant prognostic factor in GC (Zhang et al., 2014). Despite many studies of the progression of GC, the biomarkers and underlying molecular mechanisms of lymph node metastasis in GC remain unclear. Long non-coding RNAs (lncRNAs), originally were thought to be have no coding ability, but recent studies have shown that some of the RNAs, considered as non-coding, are translated into proteins (Matsumoto et al., 2017; Rion and Rüegg, 2017; Cardon et al., 2020). Previous studies have suggested that lncRNAs are engaged in the many biological processes of diseases including cancer. For instance, PVT1 promotes cancer progression in gallbladder cancer (Chen et al., 2019); NORAD promotes cell proliferation, invasion, and migration and inhibits apoptosis in lung cancer (Li et al., 2020); and MFI2-AS1 promotes cancer progression and metastasis in hepatocellular carcinoma (Wei et al., 2020). MicroRNAs (miRNAs) are a type of noncoding RNA of approximately 22 nucleotides in length. They can target the 3' untranslated regions (3' UTRs) of mRNAs, thereby silencing RNA expression at the post-transcriptional level (Kim et al., 2018; Zhang et al., 2018b). The famous competing endogenous RNA (ceRNA) hypothesis proposes that lncRNAs can act as ceRNAs to compete for miRNA response elements, thus regulating target mRNA expression (Salmena et al., 2011). The function of such a ceRNA network has been verified in many cancers, including Wilms’ tumor (Zheng et al., 2020) and cervical cancer (Chen et al., 2020). However, the survival-related ceRNA network in GC needs further study.

**Figure 1:**
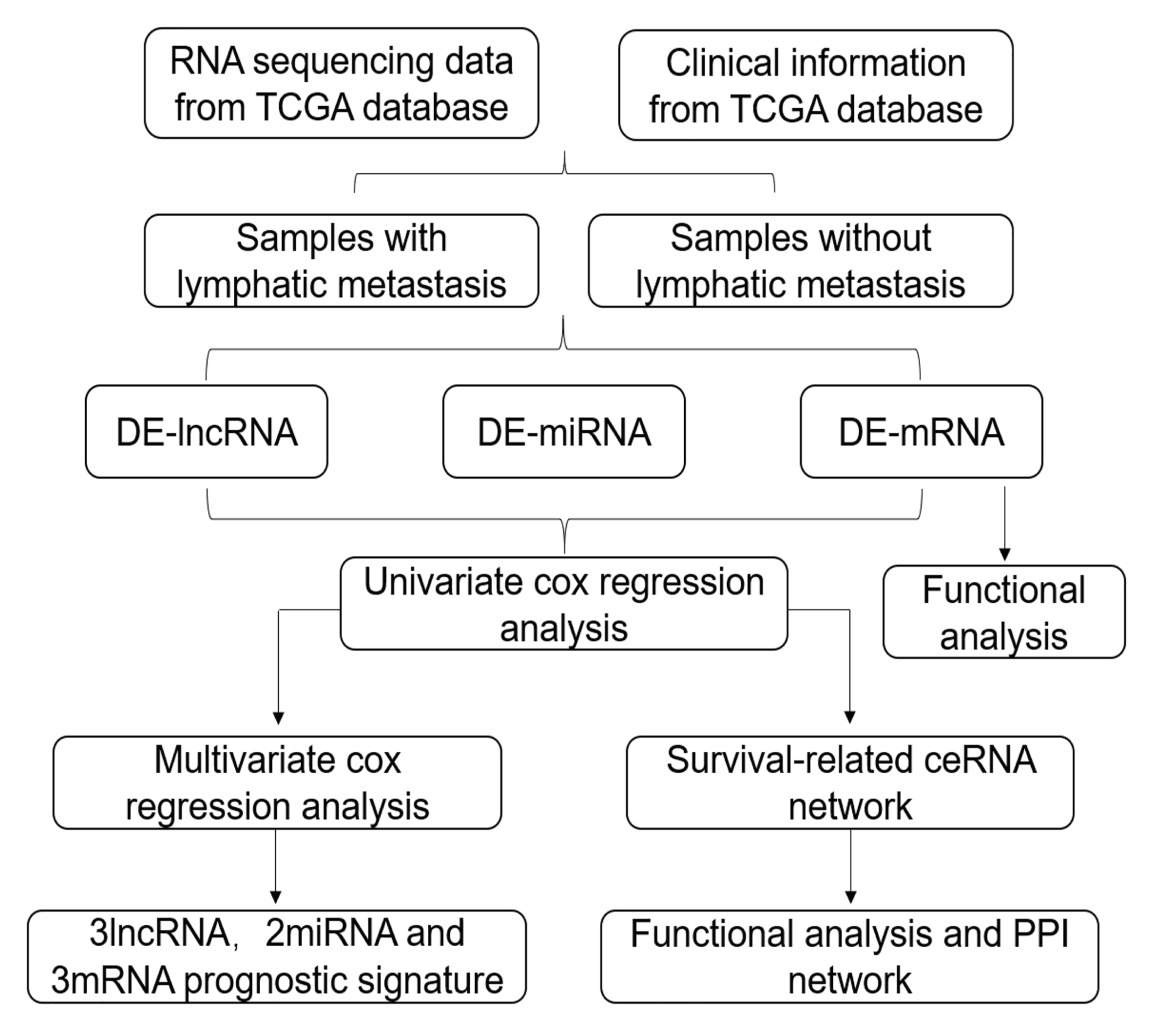
Flow chart of the analysis procedure. The chart shows the steps involved in screening the prognostic risk score model.

In this study, we obtained GC RNA sequencing data from The Cancer Genome Atlas (TCGA) to identify differentially expressed RNAs (DE-RNAs) between patients with lymphatic metastasis and those without lymphatic metastasis. Based on these DE-RNAs and the clinical information of GC patients, we screened survival-related DE-RNAs to construct a survival-related ceRNA network. In addition, we used three lncRNAs (AC094104.2, AC010457.1, and AC091832.1), two miRNAs (miR-653-5p and miR-3923), and three mRNAs (C5orf46, EPHA8, and HPR) to establish a prognostic prediction model. Figure 1 depicts the procedure used for the bioinformatics analysis, and the Table 1 shows the details of the datasets. The purpose of our study was to acquire potential biomarkers associated with prognosis of GC patients and clarify the regulatory mechanism of RNAs in GC.

**Table 1:** Information of datasets.

Datasets	Source	Application
TCGA	https://gdc.cancer.gov/access-data/gdc-data-transfer-tool	Differential RNA analysis
Ensembl	http://asia.ensembl.org/index.html	Annotation information for RNA-seq data
miRBase	http://www.mirbase.org/	Mature miRNA annotation
LncBase Predicted v.2	http://carolina.imis.athena-innovation.gr/	Target prediction
miRDB	http://www.mirdb.org/	Target prediction
miRTarBase	http://mirtarbase.cuhk.edu.cn/php/index.php	Target prediction
KEGG	clusterProfiler R package	Functional enrichment analysis
GO	Metascape	Functional enrichment analysis
String	https://string-db.org/	Protein-protein interaction analysis

## Materials and Methods

### Data Collection and Preprocessing

Clinical data for GC patients, mature miRNA datasets (the Count platform), and RNA expression profiles from TCGA were obtained using the Genomics Data Commons data transfer tool.^1^ Annotated RNA sequencing datasets were downloaded from the Ensembl database,^2^ and mature annotated miRNA datasets were downloaded from the miRBase database.^3^

### Identification of DE-RNAs

According to the clinical information and RNA expression profiles of cancer patients, a total of 209 samples from patients with lymphatic metastasis (N1, N2, N3, and N4) and 93 samples from patients without lymphatic metastasis (N0) were obtained. Similarly, based on clinical information and mature miRNA datasets, a total of 223 samples from patients with lymphatic metastasis and 104 samples from patients without lymphatic metastasis were identified. The DE-RNAs between samples from the two patient groups were determined using the edgeR package of the R software. For screening criteria, the thresholds were set as |log2(fold change)| > 0.5 and *p* < 0.05. DE-RNAs were visualized using a volcano plot. The raw count was further converted into log2 values (normalized value + 1) after normalization by edgeR for use in subsequent analysis.

### Univariate Cox Regression Analysis

Based on clinical data, the survival study included patients who were followed-up from 90 to 3,650 days. Univariate Cox regression analysis was used to determine correlations between the expression levels of RNAs and overall survival. RNAs with *p* < 0.05 were regarded as candidate prognostic markers and were used for establishing the ceRNA network. Hazard ratios (HR) for the top 10 differentially expressed lncRNAs (DE-lncRNAs), all the differentially expressed miRNAs (DE-miRNAs), and the top 10 DE-mRNAs are illustrated in forest plots.

### Establishment of Survival-Related ceRNA Regulatory Network

The ceRNA network was constructed using the survival-related RNAs. The LncBase Predicted v.2 database was used to predict relationships between DE-lncRNAs and DE-miRNAs.^4^ The miRDB (version 6.0) and miRTarBase (Release 7.0) databases were used to establish relationships between DE-miRNAs and DE-mRNAs.^5^^,^^6^ Finally, the lncRNA-miRNA-mRNA ceRNA network was constructed using the Cytoscape software (version 3.7.2).

### Functional Enrichment Analysis

Functional enrichment analysis of DE-mRNAs and survival-related DE-mRNAs was used to reveal their potential functions. To analyze the DE-mRNAs at a functional level, gene ontology (GO) analysis was performed using the Metascape online tool,^7^ and Kyoto Encyclopedia of Genes and Genomes (KEGG) analysis was conducted using the clusterProfiler R package. The survival-related DE-mRNAs were also analyzed at the functional level using Metascape. Interactive relationships among survival-related DE-mRNAs were evaluated using the STRING (version 11.0) database; those with an interaction score greater than 0.4 were considered to be significant. A protein-protein interaction (PPI) network was constructed using the Cytoscape software.

### Establishment of the Prognostic Model

Survival-related RNAs were further screened by multiple regression analysis, and those with *p* < 0.05 were used to establish lncRNA, miRNA, and mRNA signature scores according to the following formula: risk score = (β1*expression level of RNA1) + (β2*expression level of RNA2) + (β3*expression level of RNA3), where β is the regression coefficient obtained from the multivariate Cox regression model. The survivalROC R package was used to construct a time-dependent receiver operating characteristic (ROC) curve for 3-year survival, and the area under the curve (AUC) was calculated to assess prognostic performance.

### Statistical Analysis

The statistical analyses in this study were performed in the R software (version 3.6.2). The figures were drawn using GraphPad Prism 8, and the overall survival rate was evaluated *via* Kaplan-Meier (K-M) analysis. *p* < 0.05 was considered to indicate statistical significance.

## Results

### Identification of DE-RNAs

**Figure 2:**
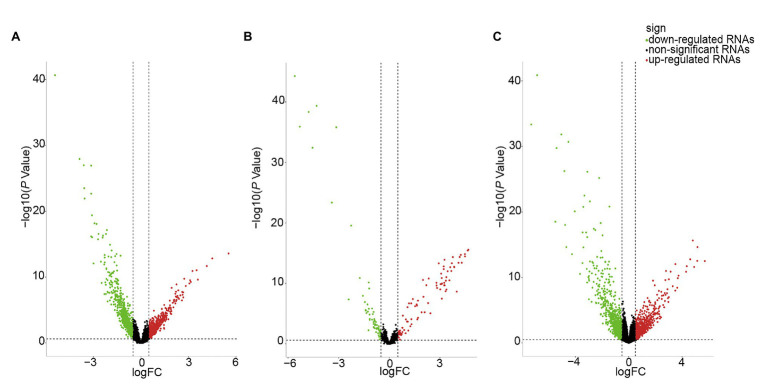
Volcano plot of differentially expressed RNAs (DE-RNAs) in lymph node metastatic gastric cancer (GC) samples and lymph node nonmetastatic GC samples. **(A)** DE-lncRNAs. **(B)** DE-miRNAs. **(C)** DE-mRNAs. Red dots indicate upregulated RNAs, green dots indicate downregulated RNAs, and black dots indicate non-differentially expressed RNAs.

We identified DE-RNAs in GC patients with and without lymphatic metastasis. As a result, 2,247 lncRNAs, 155 miRNAs, and 1,253 mRNAs were found to be differentially expressed, comprising 469 upregulated and 1,778 downregulated DE-lncRNAs (Figure 2A), 87 upregulated and 68 downregulated DE-miRNAs (Figure 2B), and 521 upregulated and 732 downregulated DE-mRNAs (Figure 2C). To further understand the functions of the DE-mRNAs, we conducted KEGG and GO analyses. The KEGG results showed that the crucial pathways included “complement and coagulation cascades,” “neuroactive ligand-receptor interaction,” and “PPAR signaling pathway” (Supplementary Figure S1A). Significant GO categories identified using the Metascape tool included “developmental process,” “biological regulation,” and “multicellular organismal process” (Supplementary Figure S1B).

### Screening of Survival-Related DE-RNAs

**Figure 3:**
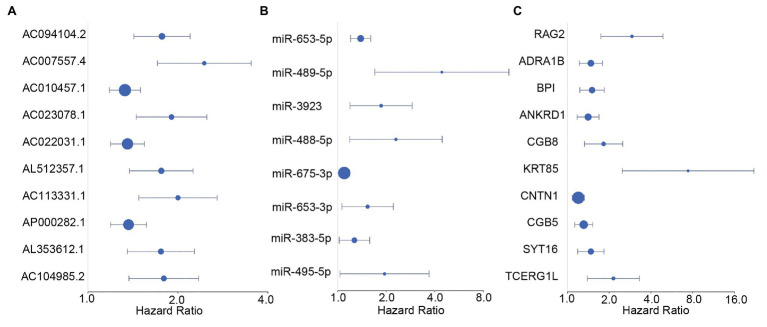
Forest plots of hazard ratios (HRs) of survival-related RNAs. **(A)** HRs of top 10 survival-related long non-coding RNAs (lncRNAs). **(B)** HRs of all significant survival-related microRNAs (miRNAs). **(C)** HRs of top 10 survival-related mRNAs.

**Figure 4:**
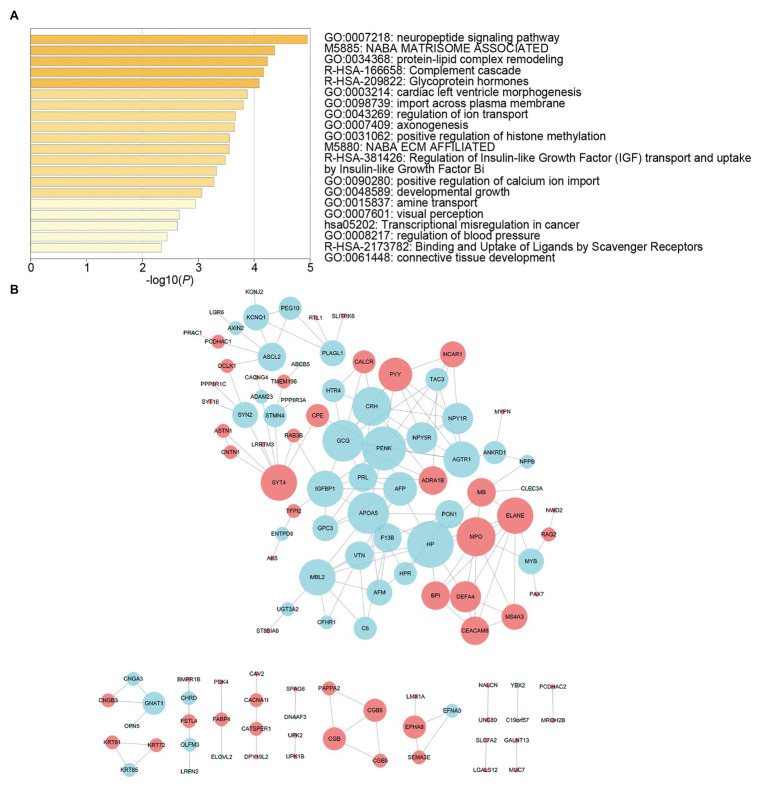
Functional enrichment analysis and protein-protein interaction (PPI) network of survival-related mRNAs. **(A)** Functional enrichment analysis of survival-related mRNAs. **(B)** PPI network of survival-related mRNAs. Red represents upregulation and blue represents downregulation. Node size is proportional to the degree of relationships among genes.

We screened survival-related DE-RNAs by univariate Cox regression analysis. As a result, 395 lncRNAs, eight miRNAs, and 180 mRNAs were found to be significantly related to patients’ overall survival time. The top 10 survival-related lncRNAs, all survival-related miRNAs, and the top 10 survival-related mRNAs are shown in Figure 3. Functional enrichment analysis using the Metascape online tool showed that these survival-related DE-mRNAs were significantly enriched in “neuropeptide signaling pathway,” “NABA matrisome associated,” and “protein-lipid complex remodeling” (Figure 4A). In addition, a PPI network of the survival-related DE-mRNAs was constructed using STRING to show the relationships among proteins. Based on the number of protein nodes forming interactions, we identified significant RNAs including HP, CRH, GCG, APOA5, and PENK (Figure 4B).

### Construction of a Survival-Related ceRNA Network

**Figure 5:**
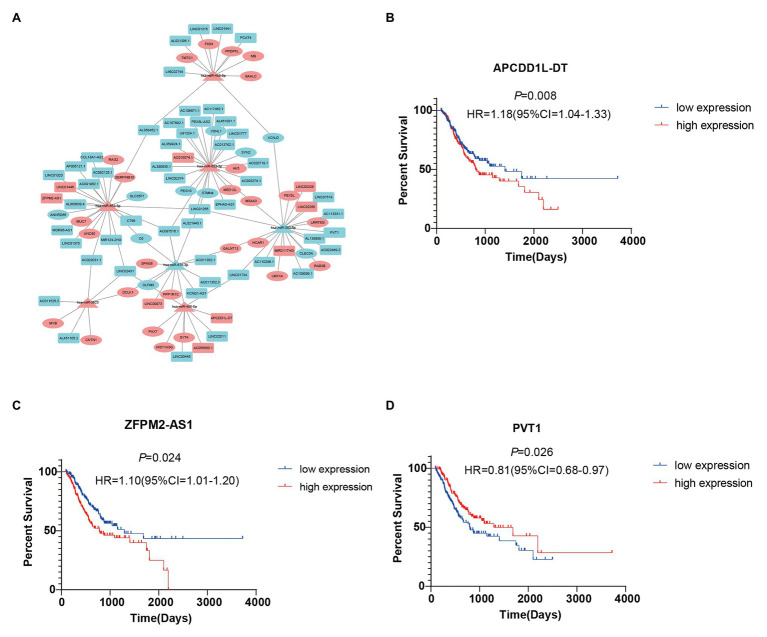
Survival-related competing endogenous RNA (ceRNA) network and Kaplan-Meier (K-M) curves for three DE-lncRNAs. **(A)** Survival-related ceRNA network in GC. Red represents upregulation and blue represents downregulation. The rectangle, triangle, and ellipse denote survival-related lncRNAs, miRNAs, and mRNAs, respectively. **(B–D)** Overall survival analysis for three DE-lncRNAs in the ceRNA network.

To further study the correlations among the survival-related DE-RNAs (lncRNAs, miRNAs, and mRNAs), we established a ceRNA network and generated a visual representation using the Cytoscape software. There were 111 interactions and 102 molecules in the network, including 59 DE-lncRNAs, seven DE-miRNAs, and 36 DE-mRNAs (Figure 5A). Furthermore, considering the significant role of lncRNAs in the ceRNA network, we selected three representative lncRNAs according to the gene expression data and determined their value in predicting prognosis using K-M analysis. Of these three DE-lncRNAs, two upregulated DE-lncRNAs (ZFPM2-AS1 and APCDD1L-DT) and one downregulated DE-lncRNA (PVT1) were associated with poor survival (Figures 5B–D).

### Construction of Prognostic Prediction Model

**Figure 6:**
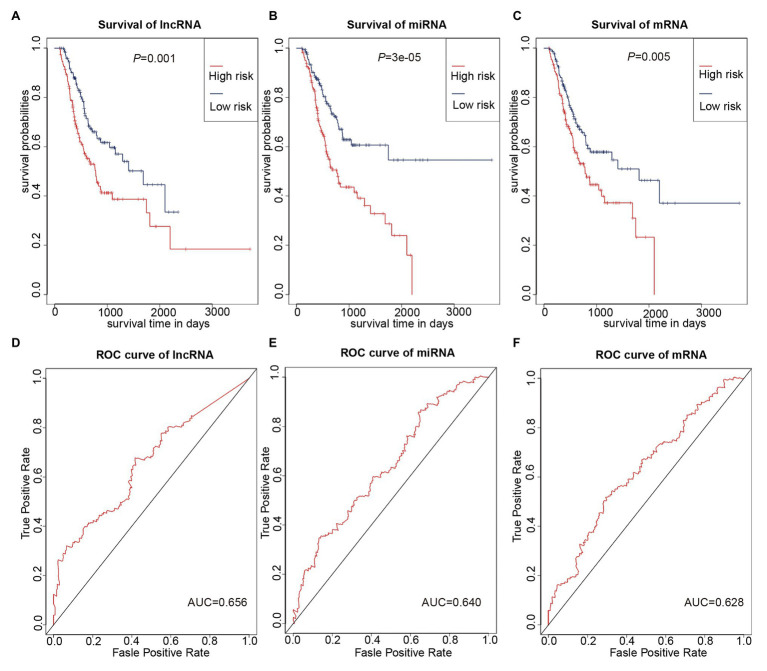
Prognostic prediction model based on three lncRNAs, two miRNAs, and three mRNAs. **(A–C)** K-M curves to assess overall survival according to high- or low-risk group based on risk scores for the three lncRNAs **(A)**, two miRNAs **(B)**, three mRNAs **(C)**. **(D–F)** Receiver operating characteristic (ROC) curves for prognostic prediction according to the risk scores of the three lncRNAs **(D)**, two miRNAs **(E)**, and three mRNAs **(F)**.

We used multivariate Cox regression analysis to further identify prognosis-related RNAs. Using *p* < 0.05 as the threshold for significance, five significant lncRNAs (Table 2), two significant miRNAs (Table 3), and nine significant mRNAs (Table 4) were found to have independent predictive value. Based on their values of *p*, the top three lncRNAs (AC094104.2, AC010457.1, and AC091832.1), all the miRNAs (miR-653-5p and miR-3923), and the top three mRNAs (C5orf46, EPHA8, and HPR) were used to establish the prognostic models. Risk score analysis was performed for the three lncRNAs, two miRNAs, and three mRNAs in each sample, and patients were divided into low- and high-risk groups according to their risk scores. K-M analysis showed that the high-risk group had worse prognoses (Figures 6A–C). The efficiencies of the above predictive models were assessed using ROC curves. The AUC values for the three lncRNAs, two miRNAs, and three mRNAs were 0.656, 0.640, and 0.628, respectively (Figures 6D–F). Thus, the risk scores could be used to evaluate the patients’ prognosis (Supplementary Figure S2).

**Table 2:** Prognosis-related lncRNAs by multivariate Cox regression analysis.

lncRNA	*p*	HR
AC094104.2	0.001	1.93
AC010457.1	0.002	1.29
AC091832.1	0.006	1.87
AL121827.2	0.015	1.14
AC110288.1	0.049	0.36

HR, hazard ratio.

**Table 3:** Prognosis-related miRNAs by multivariate Cox regression analysis.

miRNA	*p*	HR
miR-653-5p	0.001	1.46
miR-3923	0.042	1.68

HR, hazard ratio.

**Table 4:** Prognosis-related mRNAs by multivariate Cox regression analysis.

mRNA	*p*	HR
C5orf46	0.000	2.51
EPHA8	0.000	1.97
HPR	0.001	2.82
CATSPER1	0.002	0.37
MBL2	0.006	0.04
RAG2	0.011	2.66
C1QL2	0.015	1.64
ADRA1B	0.029	1.65
BPI	0.047	2.04

HR, hazard ratio.

**Figure 7:**
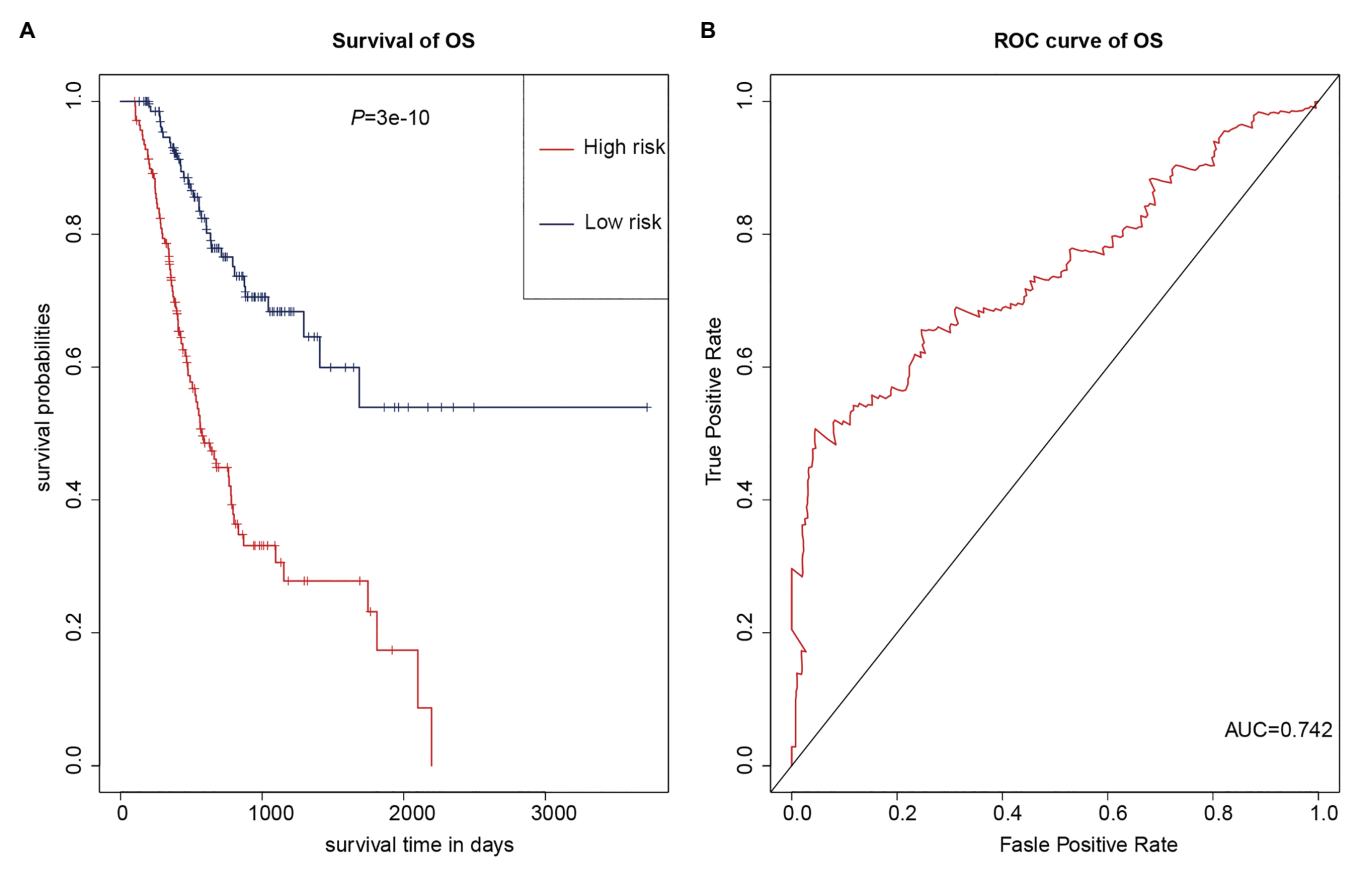
Eight-RNA prediction model. **(A)** K-M curves to assess overall survival in high- and low-risk groups according the risk scores of the eight RNAs. **(B)** ROC curves for prognostic prediction according the risk scores of the eight RNAs.

As multiple types of RNAs are thought to be involved in the occurrence and progression of cancer, we calculated risk scores using the above-mentioned eight RNAs to improve the accuracy of the prediction. The results of the K-M analysis showed that the high-risk group had worse prognoses (Figure 7A). The AUC value for the eight-RNA signature was 0.742 (Figure 7B).

## Discussion

A growing number of studies suggest that many diseases, particularly cancers, are controlled by complex gene regulation (Nibbe et al., 2011). In this study, in order to reveal the potential molecular mechanism of GC prognosis, we screened DE-lncRNAs, DE-miRNAs, and DE-mRNAs between GC patients with and without lymph node metastasis by bioinformatics analysis. Survival-related DE-RNAs were also obtained and used to construct a ceRNA network. Moreover, we used eight DE-RNAs to establish a prognostic prediction model. These results provide new insights for further research on ceRNAs in GC.

Recently, the ceRNA hypothesis was proposed as a new way for lncRNAs to regulate mRNAs by binding miRNAs competitively (Thomson and Dinger, 2016). To date, many studies have shown that lncRNAs can act as ceRNAs to regulate the development of GC; these lncRNAs include MT1JP (Zhang et al., 2018a), TMPO-AS1 (Sun and Han, 2020), and TRPM2-AS (Xiao et al., 2020). On the other hand, many miRNAs have also been shown to have important roles in the progression of GC, including miR-103a-3p (Hu et al., 2018a), miR-194 (Peng et al., 2017), and miR-4317 (Hu et al., 2018b). However, there has been limited research into the role of RNAs in lymph node metastasis in GC. In contrast with previous studies, we screened DE-RNAs between GC patients with lymphatic metastasis and those without lymphatic metastasis, rather than between the GC group and the normal group. As lymphatic metastasis may affect the prognosis of patients, DE-RNAs obtained in this way will be more representative as prognostic biomarkers for GC patients. Moreover, survival analysis of DE-RNAs was performed and, based on the identified survival-related DE-RNAs and the ceRNA theory, we established a survival-related ceRNA network containing 59 DE-lncRNAs, seven DE-miRNAs, and 36 DE-mRNAs. Some of these had been previously reported in GC, for instance, miR-383-5p was reported to be downregulated in GC tissues and cell lines (Xu et al., 2019). Moreover, downregulation of miR-383-5p promotes the development of GC and is associated with poor prognosis (Wei and Gao, 2019); miR-383-5p was also downregulated in our survival-related ceRNA network. Based on the ceRNA network, we found that C6 and DCLK1 bound to two miRNAs, respectively, and they were involved in the progression of GC. The expression level of C6 can be used to distinguish GC patients with good or poor prognosis (Yang et al., 2019), and high expression of DCLK1 predicts worse clinical outcomes in GC (Wu et al., 2020). In addition, we found that some of the RNAs had been reported in other cancers. For example, DCLK1 is not only associated with GC but also with colorectal cancer, where it can promote the epithelial-mesenchymal transition process (Liu et al., 2018b). MS4A3, which was found to bind to two miRNAs in our ceRNA network, was shown in a previous study to be a target of EVI1, and its suppression has an important role in cancer (Heller et al., 2015). However, there are still some novel RNAs that have not been reported in previous studies but were shown in our study to be related to prognosis in GC patients. Overall, we showed here for the first time that the above RNAs might be involved in regulation of the progression of GC.

To further understand the potential functions of survival-related DE-mRNAs, we performed functional enrichment analysis and constructed a PPI network. On the one hand, the enrichment analysis showed that the most significant pathway was “neuropeptide signaling pathway.” Studies have shown that the neuropeptide Y (NPY) family and its associated receptors have crucial roles in the progression of cancers (Ruscica et al., 2007). In addition, gastrin serves physiological functions as hormones in the gastrointestinal tract and as neuropeptides in the nervous system (Heasley, 2001). The gastrin-mediated signaling pathways, such as Indian Hedgehog signaling, may be related to the development of GC (Hayakawa et al., 2016). Moreover, some other pathways were associated with cancer, including “complement cascade” and “Matrisome associated.” Recent studies have shown that complement system promotes cancer development and progression (Rutkowski et al., 2010). And the complement activation enhances tumor growth and increases metastasis in the tumor microenvironment (Afshar-Kharghan, 2017). Otherwise, as is known to all, the extracellular matrix (ECM) consists of 1,100 core-matrisome and matrisome-associated proteins and of glycosaminoglycans (Ricard-Blum and Vallet, 2016). Studies have shown that ECM plays an important role in the development of gastric cancer (Moreira et al., 2020). On the other hand, we identified several hub RNAs based on the PPI network, including HP, CRH, GCG, APOA5, and PENK. Some of these genes are involved in the development of cancer, for instance, PENK is downregulated in osteosarcoma and can activate the PI3K/Akt signaling pathway to restrain osteosarcoma cell migration (Zhang et al., 2020). Notably, PENK was also downregulated in our PPI network. Moreover, APOA5 on 11q23.3 is significantly related to prostate cancer risk (Major et al., 2014). In brief, these results provide clues for further study of the mechanisms underlying GC.

In the present study, based on multivariate Cox regression analysis, we used three lncRNAs, two miRNAs, and three mRNAs to construct the prognostic prediction models. These biomarkers have rarely been reported in GC, but they have been widely studied in other cancers. For instance, miR-653-5p overexpression was found to inhibit tumor growth in melanoma (Liu et al., 2020) but to promote cell proliferation and invasion in prostate cancer (Fu et al., 2019). In pancreatic cancer, miR-3923 was shown to inhibit cell viability (Li et al., 2016), and the expression of miR-3923 was related to lymph node metastasis in breast cancer (Wang et al., 2014). EphA8 was identified as a prognostic biomarker for epithelial ovarian cancer and oral tongue squamous cell carcinoma (Liu et al., 2016, 2018a). Most importantly, EphA8 contributed to poor prognosis *via* regulation of ADAM10 in GC (Chi et al., 2019). Our models also included AC094104.2, AC010457.1, AC091832.1, C5orf46, and HPR; their molecular mechanisms in GC need further research. At present, there have been few studies of lncRNAs, miRNAs, and mRNAs related to lymph node metastasis in GC. However, considering the important role of lymph node metastasis and ceRNA regulatory networks in cancer, the RNAs identified in this study may become novel diagnostic biomarkers and therapeutic targets for GC, and provide new insights for further in-depth research on the diagnosis and treatment of GC.

In conclusion, in the present study, a survival-related ceRNA network was constructed and prognostic biomarker prediction models were established. These results provide new insights into the ceRNA network in GC and novel clues for the screening of prognostic biomarkers of GC. However, this study had several limitations: first, the results of the analysis need to be verified by other experiments; and second, the biological roles of these biomarkers in GC need to be further studied.

## Data Availability Statement

The original contributions presented in the study are included in the article/Supplementary Material, and further inquiries can be directed to the corresponding author.

## Author Contributions

YZe and YZa designed and supervised the study. HZ and QJ analyzed the data. HZ wrote the original draft of the paper. XL edited the draft. All authors contributed to the article and approved the submitted version.

### Conflict of Interest

The authors declare that the research was conducted in the absence of any commercial or financial relationships that could be construed as a potential conflict of interest.

## Footnotes

^1^https://gdc.cancer.gov/access-data/gdc-data-transfer-tool

^2^http://asia.ensembl.org/index.html

^3^http://www.mirbase.org/

^4^http://carolina.imis.athena-innovation.gr/

^5^http://www.mirdb.org/

^6^http://mirtarbase.cuhk.edu.cn/php/index.php

^7^http://metascape.org
