# Supplementary figures and images for: Comprehensive Analysis of Survival-Related lncRNAs, miRNAs, and mRNAs Forming a Competing Endogenous RNA Network in Gastric Cancer
Source: Front Genet. 2021 Mar 2;12:610501. doi: 10.3389/fgene.2021.610501 (PMC7960915; doi:10.3389/fgene.2021.610501)

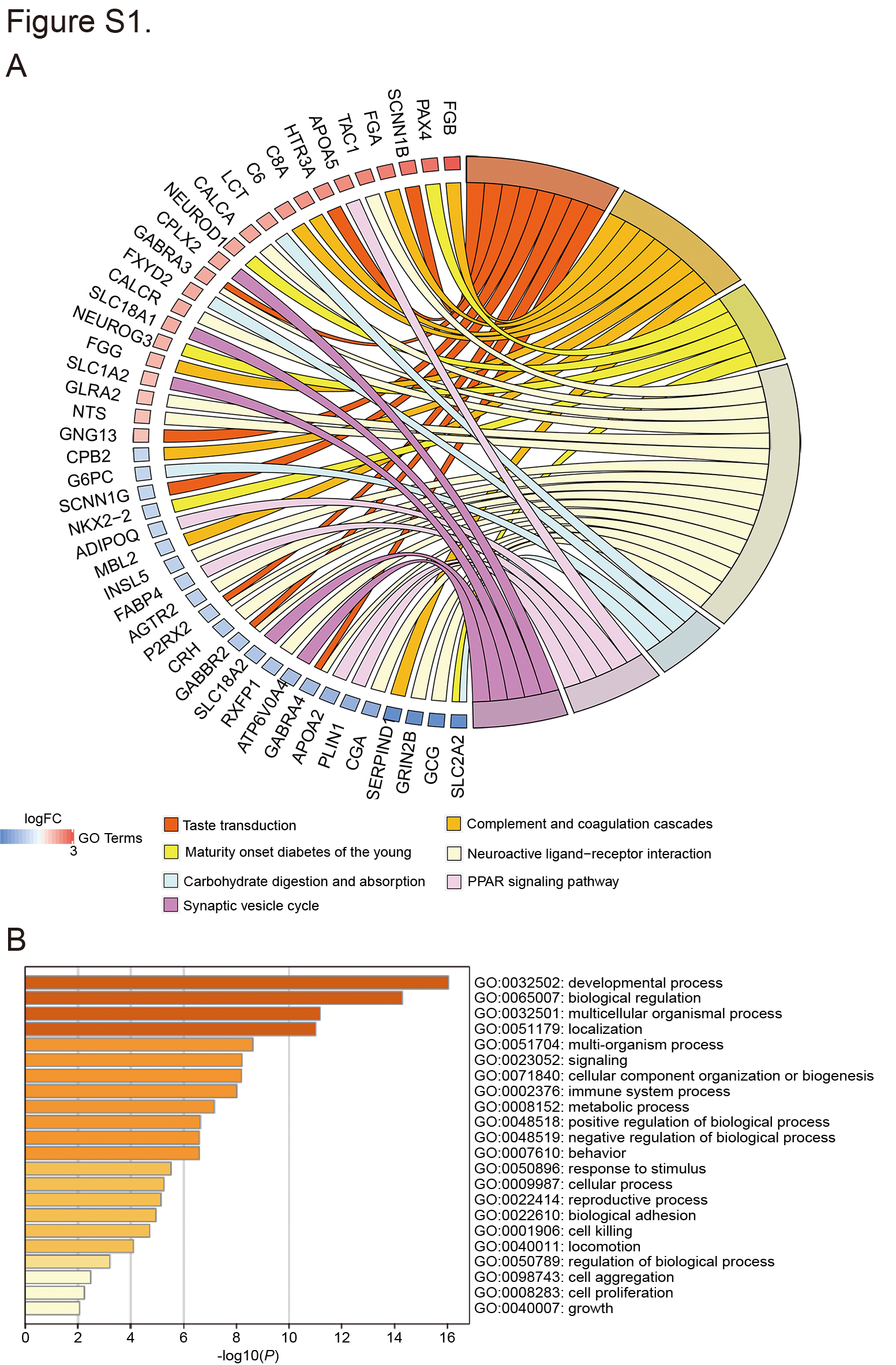

Supplement: Supplementary file 1 [file Image_1.tif]
